# IRCM‐Caps: An X‐ray image detection method for COVID‐19

**DOI:** 10.1111/crj.13599

**Published:** 2023-03-15

**Authors:** Shuo Qiu, Jinlin Ma, Ziping Ma

**Affiliations:** ^1^ School of Computer Science and Engineering North Minzu University Yinchuan China; ^2^ Key Laboratory of Intelligent Information Processing of Image and Graphics, State Ethnic Affairs Commission Yinchuan China; ^3^ School of Mathematics and Information Science North Minzu University Yinchuan China

**Keywords:** CapseNet, cascade network, CNN, COVID‐19, deep learning, X‐ray

## Abstract

**Objective:**

COVID‐19 is ravaging the world, but traditional reverse transcription‐polymerase reaction (RT‐PCR) tests are time‐consuming and have a high false‐negative rate and lack of medical equipment. Therefore, lung imaging screening methods are proposed to diagnose COVID‐19 due to its fast test speed. Currently, the commonly used convolutional neural network (CNN) model requires a large number of datasets, and the accuracy of the basic capsule network for multiple classification is limital. For this reason, this paper proposes a novel model based on CNN and CapsNet.

**Methods:**

The proposed model integrates CNN and CapsNet. And attention mechanism module and multi‐branch lightweight module are applied to enhance performance. Use the contrast adaptive histogram equalization (CLAHE) algorithm to preprocess the image to enhance image contrast. The preprocessed images are input into the network for training, and ReLU was used as the activation function to adjust the parameters to achieve the optimal.

**Result:**

The test dataset includes 1200 X‐ray images (400 COVID‐19, 400 viral pneumonia, and 400 normal), and we replace CNN of VGG16, InceptionV3, Xception, Inception‐Resnet‐v2, ResNet50, DenseNet121, and MoblieNetV2 and integrate with CapsNet. Compared with CapsNet, this network improves 6.96%, 7.83%, 9.37%, 10.47%, and 10.38% in accuracy, area under the curve (AUC), recall, and F1 scores, respectively. In the binary classification experiment, compared with CapsNet, the accuracy, AUC, accuracy, recall rate, and F1 score were increased by 5.33%, 5.34%, 2.88%, 8.00%, and 5.56%, respectively.

**Conclusion:**

The proposed embedded the advantages of traditional convolutional neural network and capsule network and has a good classification effect on small COVID‐19 X‐ray image dataset.

## Graphical abstract

This paper proposes a new model based on CNN and CapsNet, which uses CLAHE algorithm to enhance image contrast, input the preprocessed image into the network for training, and use ReLU as the activation function to adjust the parameters to make it optimal. This method combines the advantages of traditional convolutional neural network and capsule network and has a good classification effect on small COVID‐19 X‐ray image datasets.

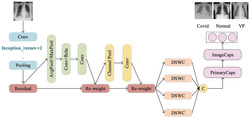

## 1 INTRODUCTION

The deep learning is used as an important means to assist the diagnosis of COVID‐19 because of its fast diagnosis speed and high accuracy. As an excellent feature extractor, convolutional neural network (CNN) can capture pixel‐level information that cannot be obviously noticed by human eyes and has been widely applied in the field of deep feature extraction,^1^, ^2^ which has been used to detect COVID‐19 by most researchers.

Although CNN has strong image processing ability, it cannot capture the spatial relationship between image instances with rotation or other transformations. Existing studies have shown that COVID‐19 has problems of insufficient datasets and data imbalance, for which researchers have put forward some solutions. Rahimzadeh and Attar^3^ used the cascade network by Xception and Resnet, which is reused COVID‐19 images in stages. The experiment was performed on 31 X‐ray images of COVID‐19, 6,851 normal and 4,420 pneumonia images, show that is achieved an average accuracy of 99.5%, and a low sensitivity of 80.5%. Afshar et al.^4^ used the method of pre training on more than 100 000 pneumonia pictures before COVID‐19 classification and finally achieved an accuracy of 98.3%. However, this method has the problem of long training time. Das et al.^5^ use transfer learning to transfer the weights, deviations, and features learned on the Imagenet dataset to the TLCoV model. The verification experiments on 219 COVID‐191345 VP and 1341 normal X‐ray images have achieved an accuracy of 97.67%.

Aiming at the problem of too little datasets, this paper uses CapsNet^6^ as the basic network to solve the defects of CNN's low recognition ability of objects after large‐scale rotation and poor spatial recognition between objects, obtain better recognition performance on small datasets, and solve the problem of dataset imbalance caused by too small COVID‐19 dataset.

In addition, many detection models based on CapsNet have the problems of good binary classification and poor Multi‐Class Classification. The evolutionary CapsNet model proposed by Toraman et al.^7^ achieved 97.24% accuracy in binary classification, but only 84.22% accuracy in three‐class classification. The VGGCapsNet model proposed by Tiwari and Jain^8^ has achieved good performance on small datasets, but the accuracy of binary classification of the network has reached 97%, whereas the accuracy of three‐class classification is only 92%. The DenseCapsNet model proposed by Quan et al.^9^ reduces the dependence of CNN on a large amount of data, but the three‐class classification accuracy of this method is only 90.7%. In order to solve the above problems, we have improved the convolution layer of CapsNet and introduced a new module structure, so that the network model can achieve better results in binary classification and Multi‐Class classification. The key points of this paper are as follows:

1. A deep learning framework IRCM‐Caps for rapid diagnosis of COVID‐19 is proposed, which is based on the CapsNet and CNN.
2. The convolution attention module is used to refine the feature map to improve the network performance.
3. The multi‐branch lightweight module (MBL) is used to reduce the network parameters.

## 2 IRCM‐Caps

IRCM‐Caps includes CNN, convolutional attention module (CBAM), MBL and capsule layer (Capsule), and its structure is shown in Figure S1. The CNN extracts the initial feature map of the X‐ray image, the CBAM enhances the features of the initial feature map, the MBL reduces the redundant information of the feature map, and the capsule layer performs classification and outputs the classification results.

### 2.1 CNN

IRCM‐Caps uses CNNs to extract the initial features of lung X‐ray images. In this paper, we investigate representative CNNs: VGG,^9^ Inception,^10^ Xception,^11^ ResNet,^12^ Inception‐ResNet,^13^ DenseNet,^14^ and MobileNet,^15^ to select the best performance among them. VGG can reduce network parameters and capture more details while ensuring the same receptive field. Inception maintains the sparsity of the network structure by gathering highly correlated features. In addition, it selects 1 * 1 convolution to reduce the amount of parameters and deepen the network. Xception has a linear stack of deeply separable convolutional layers with residual connections, which is easy defined and modified the deep network structure. Meanwhile, ResNet only needs to learn new features on the basis of previous layer features, which effectively avoids the disappearance of too small gradient information and alleviates the problems of gradient dispersion and network degradation. Inception‐ResNet is a combination of Inception module and ResNet module, which greatly improves the performance of the model. DenseNet extracts compact and distinct features through cross‐layer connections of different lengths, which effectively alleviates the problem that deep networks are difficult to optimize due to the disappearance of gradients, and finally improves the robustness of the model. MobileNet uses 3 * 3 and 1 * 1 convolutions, bottleneck operations, and average pooling to reduce the amount of parameters.

### 2.2 CBAM

Aiming at the low accuracy of CapsNet and the congestion of capsules caused by the weak ability of feature description, this paper uses the attention mechanism to refine the feature description and guide the network to focus on the key information. The CBAM^17^ is located between the CNN and the capsule network. CBAM is composed of a channel attention module and a spatial attention module.

#### 2.2.1 Channel attention module

Each channel of the feature represents a special detector, so the channel pays attention to the meaningful features. The channel attention module creates a channel attention map by using the feature relationship between channels and uses global average pooling and maximum pooling to summarize spatial features. The specific process is as follows: First, the input feature F (H × W × C) is respectively global average pooling, and the global maximum pooling is respectively performed to obtain two sizes of 1 × 1 × C characteristic channel. Then, they are sent to a two‐layer neural network. The number of neurons in the first layer is C/R (R is the reduction rate), and the number of neurons in the second layer is C. These two layers of neural networks are shared. Then, the weight coefficient MC is obtained through the sigmoid activation function after adding the obtained two features. Finally, the new scaled feature can be obtained by multiplying the input feature F by the weight coefficient.

#### 2.2.2 Spatial attention module

The spatial attention module filters the spatial features of the image. The process is as follows: First, the input feature F (H × W × C) is respectively global average pooling, and the global maximum pooling is respectively performed to obtain two sizes of H × W × 1 characteristic channel. Then, after 7 × 7 convolution layers and Sigmoid activation function to obtain the weight coefficient MS. Finally, the new scaled feature can be obtained by multiplying the input feature F by the weight coefficient.

The CBAM makes the network more convenient and efficient, which is fully proved by the network module effectiveness experiment in Section 4.2.

### 2.3 MBL

In order to eliminate the influence of the increase of CBAM parameters on the calculation performance of the model, the MBL is added after CBAM. MBL is composed of multiple parallel branches composed of deep separable convolution.4.2 Network module effectiveness experiments show that the number of parameters in the model with MBL is reduced by about 30% compared with the model without MBL.

#### 2.3.1 Depthwise separable convolution

Depthwise separable convolution^18^ can reduce the number of parameters of the model. Its extraction process is divided into two steps.

Step 1: Perform a convolution operation on each channel in the target region of the input image.
Step 2: Use 1 × 1 convolution kernel to perform a standard convolution operation on the result obtained in the first step and change the number of channels. This step is also an important measure for DSWC to reduce the number of parameters when performing convolution operations.

Suppose the input feature map size is $M_{i}\times M_{i}$, the number of channels is L, the convolution kernel size is $N_{f}\times N_{f}$, and the number of convolution kernels is N. The parameter quantities of standard convolution (ISC) and DSWC (IDC) are calculated as follows:

Isc=Mi*Mi*L*N*Nf*Nf.

$$I_{DC}=\mathrm{Mi}*Mi*L*Nf*Nf+N*L*Nf*Nf.$$

It can be seen from the above formula that when the convolution operation is performed, when the number of convolution kernels is greater than one, the standard convolution has more parameters than DSWC. In this paper, DSWC is used to significantly reduce the number of parameters and training time of the model.

#### 2.3.2 Multi‐branch parallelism

MBL is realized by adding maximum pooling and residual connections on the basis of the Inception structure. First, the module parallelizes the depth‐separable convolution of four different receptive fields to extracts multi‐scale features to improve the adaptability of the network to different scales. Add the output features are aggregated with strong correlation, decompose the sparse distribution features into multiple dense distribution subsets which can reduce redundant information, and effectively expand the depth and width of the network. Then, max pooling and average pooling with a stride of two are used to reduce the size of the feature map and reduce the dimensionality. And fewer parameters can prevent the network from overrunning. Finally, residual connection is utilized to improve the parameter transfer efficiency and alleviate the gradient dispersion problem. Thus, the feature learning and model expression ability are improved by increasing the network depth, which makes the model easy to train.

### 2.4 CapsNet

Unlike CNNs, CapsNet constructs shallow network and employs capsule layers in other layers, thus avoiding the need for deeper networks. Each capsule is used to detect a specific entity in the image, and thus, a dynamic routing mechanism sends the detected entity to the parent layer. Compared with CNNs, which require thousands of images to be considered in many aspects, capsule networks can recognize objects from multiple angles in different situations, which can reduce the dependence on the amount of data.

## 3 EXPERIMENT

### 3.1 Dataset

The experiments use two datasets, the COVID‐19 adiography database and Chestxray.

The COVID‐19 adiography database is a large open COVID‐19 dataset, containing 3616 COVID‐19 positive, 10 192 normal, 6012 Lung opacity (non‐COVID‐19), and 1345 VP images (source: https://www.kaggle.com/tawsifurrahman/covid19-radiography-database).

Chestxray is a publicly available pneumonia case dataset, containing a total of 5856 X‐ray images, mainly classified into pneumonia and normal (source: https://www.kaggle.com/andrewmvd/pediatric-pneumonia-chest-xray).

This paper mainly conducts three‐classification and binary‐classification experiments based on the above datasets. Table 1 listed the composition of the datasets. The three‐classification data are mainly from COVID‐19, VP images from the COVID‐19 adiography database, and normal images from Chestxray. The binary data are mainly from COVID‐19 and VP images of COVID‐19.

**TABLE 1:** Datasets.

Dataset	Type	COVID‐19	Normal	VP	Total
Three classification	Train	190	190	190	570
	Test	150	150	150	450
	Val	60	60	60	180
	Total	400	400	400	1200
Two classification	Train	190	‐	190	380
	Test	150	‐	150	300
	Val	60	‐	60	120
	Total	400	‐	400	800

### 3.2 Preprocessing

This paper uses CLAHE^19^ to enhance the image contrast, and CLAHE obtains more details of the image by improving the local contrast of the image. CLAHE clips the histogram with a pre‐defined threshold to change the slope of the cumulative histogram (CDF), which, in turn, affects the slope of the transform function to achieve the purpose of contrast clipping.

The more blocks the image is segmented into, the more accurate the CLAHE processing, and the better the detail processing. After many experiments, considering the operation speed and processing power, we selected the parameters clipLimit = 2.5, and tileGridSize is (8,8). After CLAHE preprocessing, X‐ray image details are more prominent, lung contour is clearer, and the histogram is more balanced.

The three‐classification experiments before and after image enhancement in the dataset show that the images processed by the CLAHE algorithm are beneficial to the classification effect. The experimental results before and after image preprocessing are shown in Table 2.

**TABLE 2:** Model performance before and after CLAHE pretreatment.

	ACC	AUC	PRE	REC	F1
Original	0.9215	0.9117	0.8929	0.8822	0.8828
Enhance	**0.9304**	**0.9217**	**0.9123**	**0.8956**	0.8960

*Note*: Bold font is the optimal value for each column.

Abbreviation: AUC, area under the curve.

### 3.3 Evaluation indicators

We utilize seven metrics: Accuracy, Receiver Operating Characteristic Curve, Sensitivity, Specificity, Precision, Recall, and F1 score. The calculation formula is as follows:

(1) $$\text{Accuracy}=\frac{\mathrm{TP}+\mathrm{TN}}{\mathrm{TP}+\mathrm{FP}+\mathrm{FN}+\mathrm{TN}},$$

(2) $$\begin{array}{c} \mathrm{FPR}=\frac{\mathrm{FP}}{\mathrm{FP}+\mathrm{TN}} \end{array},$$

(3) $$\begin{array}{c} \mathrm{TPR}=\frac{TP}{TP+FN}, \end{array}$$

(4) $$\begin{array}{c} \mathrm{SEN}=\frac{\mathrm{TP}}{\mathrm{TP}+\mathrm{FN}} \end{array},$$

(5) $$\begin{array}{c} \mathrm{SPE}=\frac{\mathrm{TN}}{\mathrm{TN}+\mathrm{FP}} \end{array},$$

(6) $$\begin{array}{c} \text{Precision}=\frac{\mathrm{TP}}{\mathrm{TP}+\mathrm{FP}}, \end{array}$$

(7) $$\begin{array}{c} \text{Recall}=\frac{\mathrm{TP}}{\mathrm{TP}+\mathrm{FN}}, \end{array}$$

(8) $$\begin{array}{c} F1=2\times \frac{\text{Precision}\times \text{Recall}}{\text{Precision}+\text{Recall}}, \end{array}$$

where TP is the number of positive examples that predicted correctly, TN is the number of negative examples that predicted correctly, FP is the number of negative examples that predict incorrectly, and FN is the number of positive examples that predict correctly. The ROC curve describes the degree of change between the true positive rate (TPR, ordinate) and the false positive rate (FPR, abscissa). TPR refers to the probability that it is actually predicted under that category. FPR denotes to the probability that the prediction is correct under all categories other than this. Area under the curve (AUC) is the area under the ROC curve. The closer the AUC is to 1, the higher the authenticity of the test method.

### 3.4 Parameter setting

In this paper, the stochastic gradient descent (SGD) optimizer is used for optimization. Different network models are trained on the same dataset with the same parameters, and the training is stopped when a fixed period is reached, and finally, the weight is selected when the loss is stable.

The SGD optimizer randomly selects a sample for training and gradient update each time. In order to ensure that the model parameters are updated quickly and converge to the global optimal point, an exponentially decaying learning rate is used; that is, t the learning rate is reduced by 1/10 every 10 batches during training, and the learning rate decay value decay after each update is set to 1e‐4. In order to slow down the oscillation degree of gradient descent and speed up the convergence, a momentum of 0.9 is used in each calculation gradient, and the optimized gradient is the exponentially weighted average of the gradient from the start time to the current time. Different classification tasks use different initial learning rates and batch sizes. For the three‐class classification, the learning rate is 0.0001, and the batch size is 32; for the binary classification, the learning rate is 0.0001, and the batch size is 16.

## 4 EXPERIMENT AND RESULT ANALYSIS

In order to verify the classification effect of the model, this paper mainly conducts basic network selection experiments, network module validity experiment, and algorithm comparison experiments. The datasets are three‐class classification (COVID‐19, VP and normal) and binary classification (COVID‐19 and VP) datasets.

### 4.1 Comparison of CNNs

This experiment adopts VGG, Inception, Xception, ResNet, Inception‐ResNet, DenseNet, and MobileNet, as the basic convolutional network, and CBAM, MBL, and capsule network to form seven different classification models, which are, respectively, represented as:

VGG_CBAM_MBL‐Caps (VCM‐Caps),
Inception_CBAM_MBL‐Caps (ICM‐Caps),
Xception_CBAM_MBL‐Caps (XCM‐Caps),
Resnet_CBAM_MBL‐Caps (RCM‐Caps),
Inception‐ResNet_CBAM_MBL‐Caps (IRCM‐Caps),
DenseNet_CBAM_MBL‐Caps (DCM‐Caps),
MobileNet_CBAM_MBL‐Caps (MCM‐Caps).

To avoid the good effect of binary classification and poor effect of three‐class classification, this experiment mainly conducts experiments on three‐class classification. Experiments compare the classification capabilities of these models to choose the best base convolutional network. Tables 3 and 4 show the experimental results of three‐class classification and binary classification, respectively.

**TABLE 3:** Comparison of classical experiments in three categories.

Network structure	ACC	AUC	PRE	REC	F1	Parameter
VCM‐Caps	0.9585	0.9533	0.9430	0.9378	0.9379	20 473 940
ICM‐Caps	0.9867	0.9849	0.9801	0.9800	0.9799	25 767 740
XCM‐Caps	0.9570	0.9517	0.9402	0.9356	0.9357	25 767 740
RCM‐Caps	0.9793	0.9767	0.9707	0.9689	0.9690	28 493 972
DCM‐Caps	0.9881	0.9867	0.9822	0.9822	0.9821	8 448 340
MCM‐Caps	0.9733	0.9700	0.9613	0.9600	0.9596	**4 346 068**
IRCM‐Caps	**0.9911**	**0.9900**	**0.9866**	**0.9867**	**0.9866**	57 233 140

*Note*: Bold font is the optimal value for each column.

Abbreviation: AUC, area under the curve.

**TABLE 4:** Comparison of classical experiments in two categories.

Network structure	ACC	AUC	PRE	REC	F1	Parameter
VCM‐Caps	0.9733	0.9733	0.9863	0.9600	0.9730	20 473 940
ICM‐Caps	0.9833	0.9833	**1**	0.9667	0.9830	25 767 740
XCM‐Caps	0.9667	0.9667	0.9545	0.9800	0.9671	25 767 740
RCM‐Caps	0.9800	0.9800	0.9865	0.9733	0.9799	28 493 972
DCM‐Caps	0.9867	0.9867	**1**	0.9667	0.9830	8 448 340
MCM‐Caps	0.9700	0.9700	0.9796	0.9600	0.9697	**4 346 068**
IRCM‐Caps	**0.9900**	**0.9900**	**1**	**0.9800**	**0.9898**	57 233 140

*Note*: Bold font is the optimal value for each column.

Abbreviation: AUC, area under the curve.

As can be seen from Tables 3 and 4, IRCM‐Caps achieved similar results in three‐class classification and binary classification. On the same dataset, the values of accuracy, ROC, precision, recall, and F1 of IRCM‐Caps composed of the Inception‐ResNet‐V2 CNN are 99.11%, 99%, 98.66%, 98.67%, and 98.66%, respectively. All the five indexes are higher than other classification models composed of basic convolutional networks. The classification model based on MoblieNetV2 convolutional network is lower than IRCM‐Caps in accuracy, ROC, precision, recall, and F1, but its parameter quantity is about 7.5% of IRCM‐Caps, and it shows that lightweight is our main research direction in the future.

### 4.2 Experiment of network module effectiveness

The model in this paper is mainly composed of four parts: CNN, CBAM, MBL module, and capsule layer. In order to verify the effectiveness of each module or component, ablation experiments are performed on the three‐class classification datasets, and the results are shown in Table 5. From Table 5, it can be found that the accuracy of IR‐caps is 5.63% higher than that of CapsNet, indicating that adding a CNN can effectively improve the classification accuracy; IRC‐caps increases AUC by nearly 1% by adding a CBAM module; as the addition of modules increases the number of network parameters, MBL module is added in this paper. Because MBL possesses the advantages of the depthwise separable convolution and Inception, the IRCM‐Caps network with the MBL module improves the performance while reducing the amount of parameters by about 30% compared to IRC‐caps.

**TABLE 5:** Ablation experiment.

Network structure	ACC	AUC	PRE	REC	F1	Parameter
CapsNet	0.9304	0.9217	0.9123	0.8956	0.8960	**266 304**
IR‐caps	0.9778	0.9750	0.9672	0.9667	0.9666	79 515 104
IRC‐caps	0.9851	0.9833	0.9780	0.9778	0.9777	81 876 722
IRCM‐Caps	**0.9911**	**0.9900**	**0.9866**	**0.9867**	**0.9866**	57 233 140

*Note*: CapsNet is the basic network; IR‐caps adds a convolutional neural network on the basis of CapsNet; IRC‐caps adds CBAM module on the basis of IR‐caps; IRCM‐Caps adds MBL on the basis of IRC‐caps Module.; bold font is the optimal value for each column.

Abbreviation: AUC, area under the curve.

Minimizing false‐negative and false‐positive results is important in medical research, especially for image analysis of critical diseases such as COVID‐19. The false negatives and false positives of the four models can be clearly seen from the confusion matrix in Figure S1. In the figure, the vertical axis is the real label, the horizontal axis is the predicted label, 0 represents COVID‐19, 1 represents normal, and 2 represents VP. It can be seen that CapsNet has a high classification accuracy for normal, and it is easy to misjudge COVID‐19 and VP as normal. The misjudgment situation is improved with the addition of CNN, CBAM, and MBL modules. Among these adding strategies, IRCM‐Caps has the highest classification accuracy for the three types, and each type of samples is balanced, indicating that this method can achieve better overall classification, stronger network feature extraction ability, and achieve good generalization. However, there is still a small amount of classification errors due to the too similar regions of COVID‐19 and VP lesions.

The ROC curve is described by plotting the TPR and FPR in a graph with various threshold settings, which is benefit to classify, analyze, and visualize the classification results. The ROC curves on the three‐classification datasets are shown in Figure S1. It can be observed that with the increase of the number of iterations, the error rate gradually decreases, in which there is no over‐fitting phenomenon, and the overall curve is located in the upper left corner, indicating that IRCM‐CAPS has good performance. The ROC area of the four experiments shows an increasing trend, among which the micro average area and macro average area of the ROC curve of IRCM‐Caps both reached 0.99, which demonstrates the effect was the best. Meanwhile, this shows that the method is more stable and robust. In addition, CapsNet and IR‐CAPS have lower ability to classify VP than COVID‐19 and normal, and IRC‐CAPS has lower ability to classify COVID‐19 and VP than normal, whereas IRCM‐Caps has good discrimination ability for all categories. It can better learn and identify lesion features with large spatial scale differences.

### 4.3 Compared with CapsNet‐based model

This section compares the performance of IRCM‐Cap with other CapSnet‐based methods in terms of dataset, accuracy, specificity, sensitivity, and precision, as shown in Table 6. COVID‐FACT^20^ requires fewer training parameters but is easily misclassified as COVID‐19. COVID‐CAPS^4^ address the dataset imbalance, but the number of pre‐trained lung images is huge. The Convolutional CapsNet^7^ model is relatively simple, but the dataset used is large and requires a lot of time and hardware resources to process the images. Relatively speaking, although IRCM‐Cap has a large number of parameters; it has certain advantages because of its high accuracy and less required datasets.

**TABLE 6:** Compared with CapsNet‐based model.

Method	Dataset	ACC	SEN	PRE	SPE
IRCM‐Cap	450 COVID‐19, 450 VP, 450 normal	**0.9911**	**0.9888**	0.9866	0.9845
	450 COVID‐19, 450 VP	0.9900		**1**	
COVID‐FACT^20^	COVID‐CT‐MD	0.9082	0.9455		0.8604
COVID‐CAPS^4^	112 120 X‐ray images	0.9830			**0.9860**
Convolutional CapsNet^7^	231 NCP, 1050 normal, 1050 pneumonia	0.8422			
	231 NCP, 1050 normal	0.9724			

*Note*: Bold font is the optimal value for each column.

### 4.4 Compared with multi‐network fusion model

This section compares the performance of IRCM‐Cap with other methods based on multi‐network fusion in terms of dataset, accuracy, specificity, sensitivity, and precision, as shown in Table 7. ResNet50 + Xception^3^ has the best accuracy and specificity, but it has low sensitivity and requires a large number of datasets. DenseCapsNet^21^ reduces the reliance of CNN on large amounts of data but has low accuracy. COFE‐Net uses fuzzy measurement to greatly reduce the search space, but it takes a long time and will cause certain classification errors. VGGCapsNet^8^ solves the limitations of traditional CNN and enhances the computing power of the initial feature map, but the effect of multiple classification is not good. In summary, IRCM‐Cap is the best model with high indexes.

**TABLE 7:** Compared with CapsNet‐based model.

Method	Dataset	ACC	SEN	PRE	SPE
IRCM‐Cap	450 COVID‐19, 450 VP, 450 normal	0.9911	**0.9888**	0.9866	0.9845
	450 COVID‐19, 450 VP	0.9900		**1**	
ResNet50 + Xception^3^	8851 normal, 6012 pneumonia, Chestxray	**0.9950**	0.8050		**0.9960**
DenseCapsNet^21^	750 X‐ray images	0.9070	0.9600		
COFE‐Net^9^	568 NCP, 6052 pneumonia, 8851 normal	0.9830		0.9840	
VGGCapsNet^8^	219 NCP, 1345 pneumonia, 1341 normal	0.9200			
	219 NCP, 1345 pneumonia	0.9700			

*Note*: Bold font is the optimal value for each column.

### 4.5 Comparison with state‐of‐the‐art approaches

This experiment compares the performance of IRCM‐Caps and SoTA methods in terms of dataset, accuracy, specificity, sensitivity, and precision. The experimental results are shown in Table 8. SOTA methods include DeCoVNet,^21^ VSBN,^22^ COVNet,^23^ ResNet50,^24^ COVID‐Net,^25^ DenseNet121,^26^ SVM,^27^ VGG19,^28^ DLM,^29^ HTV^30^ (homomorphic transformation and VGG), ChestX‐ray6,^31^ and Dense‐CNN.^32^

**TABLE 8:** Comparison of classic papers.

Method	Dataset	ACC	SEN	PRE	SPE
IRCM‐Cap	450 COVID‐19, 450 VP, 450 normal	**0.9911**	0.9888	0.9866	0.9845
	450 COVID‐19, 450 VP	**0.9900**		**1**	
DeCoVNet^22^	313 COVID‐19, 229 non‐COVID‐19	0.9590	0.9070		0.9110
VSBN^23^	125 COVID‐19, 123 CAP, 134 SPT, 139 normal	0.95 above			0.95 above
COVNet^24^	1292 COVID‐19, 1735 CAP, 1325 normal	0.9600	0.900		0.9600
ResNet50^25^	341 COVID‐19, 2772 BP, 1493 VP, 2800 normal	0.9610		0.765	0.9660
COVID‐Net^26^	COVID‐chestxray, pneumonia‐chestxray	0.8350	1	0.800	
DenseNet121^26^	924 COVID‐19, 342 non‐COVID‐19	0.8700	0.8000		
SVM^28^	53 CT images	0.9827	0.9893		0.9760
VGG19^29^	102 COVID‐19, 118 normal	0.9500	0.9600	0.9600	
DLM^30^	1252 COVID‐19 and 1230 non‐COVID‐19	0.9891	**0.9896**	0.9888	**0.9886**
HTV^31^	2250 COVID‐19, 2250 pneumonia, 2250 normal	0.9656	0.9514		
ChestX‐ray6^32^	9514 X‐ray images	0.9794			
Dense‐CNN^32^	273 COVID‐19 and 225 non‐COVID‐19	0.9378	0.9340	0.9510	0.9420

*Note*: Bold font is the optimal value for each column.

By comparison, IRCM‐CAPS shows good results in each of the performance indicators. Although individual indicators are slightly lower than those of other networks, overall, IRCM‐CAP performs well on small datasets, which can help radiologists more accurately locate the location of suspected lesions greatly reduces the pressure on doctors to deal with the epidemic.

## 5 CONCLUSION

This paper designs a model IRCM‐Caps to detecting COVID‐19. The IRCM‐Caps model is composed of CNN, CBAM, MBL, and capsule. The CNN enhances the expression ability of the initial feature map. CBAM makes the image features more prominent. MBL improves the model performance while reducing the amount of parameters. Capsule avoids increasing the convolution depth to improve the network performance. Comparative experiments show that IRCM‐Caps has better accuracy than CapsNet; IRCM‐Caps has better overall performance than SOTA method. However, the parameter quantity of IRCM‐Caps is much larger than that of CapsNet, so reducing the parameter quantity of the model will be a major research direction in the future.

## AUTHOR CONTRIBUTIONS

**Shuo Qiu:** Method design; writing and editing. **Jinlin Ma:** Supervisor; writing and editing. **Ziping Ma:** Writing and editing. All authors have read and approved the manuscript.

## CONFLICT OF INTEREST STATEMENT

The authors have declared no conflict of interest.

## ETHICS STATEMENT

Not applicable.

## Supporting information


**Figure S1.** Supporting Information

## Data Availability

The data that support the findings of this study are available in COVID‐19 adiography database at weizhi. These data were derived from the following resources available in the public domain: COVID‐19 adiography database, https://www.kaggle.com/tawsifurrahman/covid19%2Dradiography%2Ddatabase ‐ Chestxray, https://www.kaggle.com/andrewmvd/pediatric%2Dpneumonia%2Dchest%2Dxray.
