# Supplementary material for: IRCM‐Caps: An X‐ray image detection method for COVID‐19
Source: Clin Respir J. 2023 Mar 15;17(5):364–73. doi: 10.1111/crj.13599 (PMC10214581; doi:10.1111/crj.13599)
Supplement: Supplementary file 1 — Figure S1. Supporting Information [file CRJ-17-364-s001.docx]

# Supplementary figures

**diagram of module architecture**


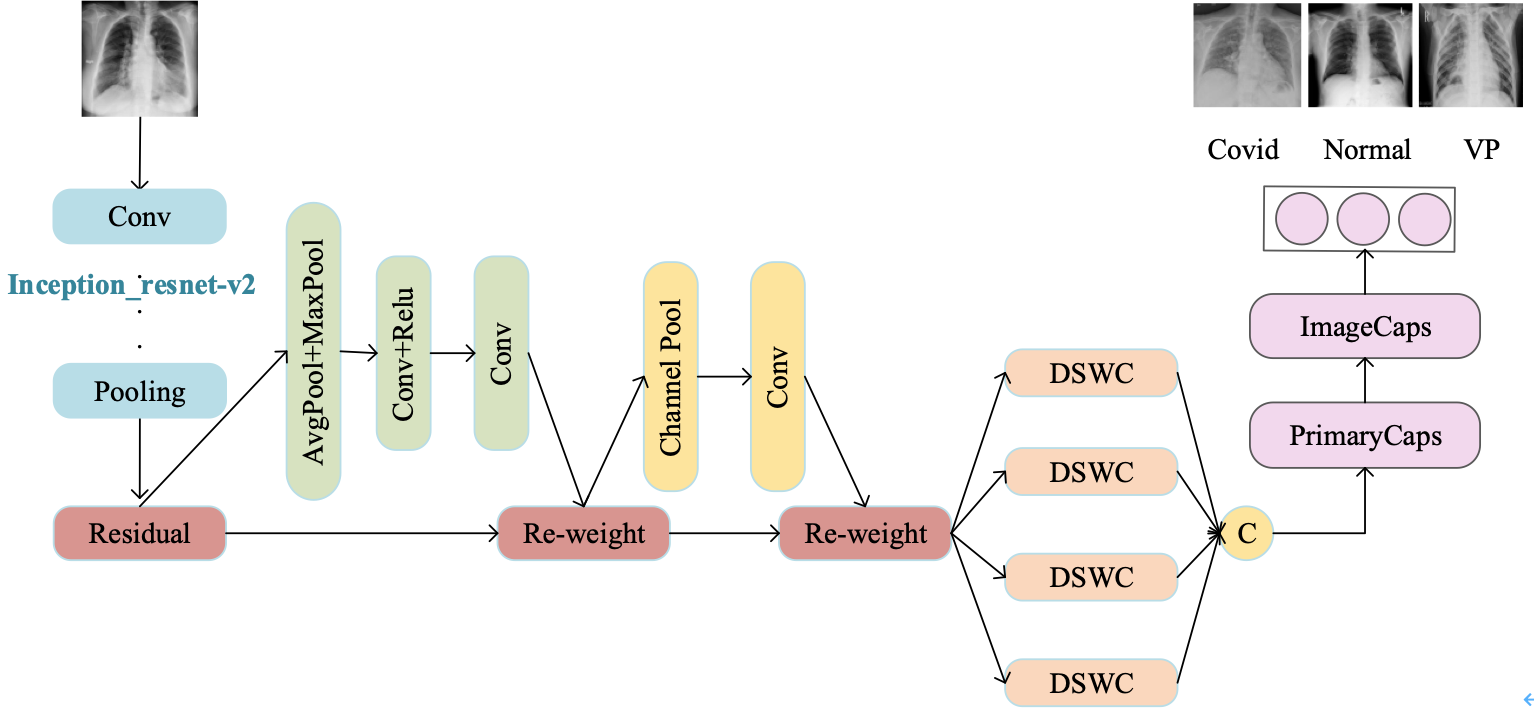


**Original and enhanced X-ray images of the lungs:**

| 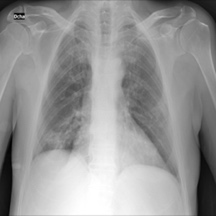 | 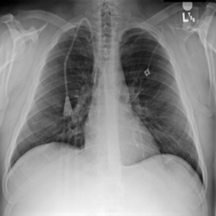 | 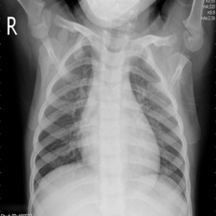 |
| --- | --- | --- |
| COVID-19 | normal | VP |
| 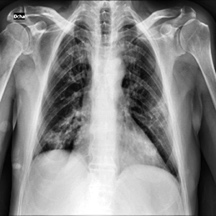 | 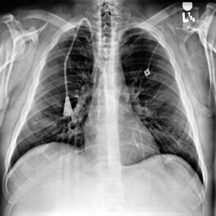 | 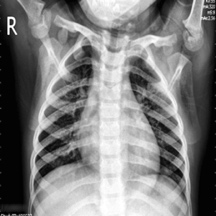 |
| Enhanced COVID-19 | Enhanced normal | Enhanced VP |

**Confusion matrix comparison diagram**

| 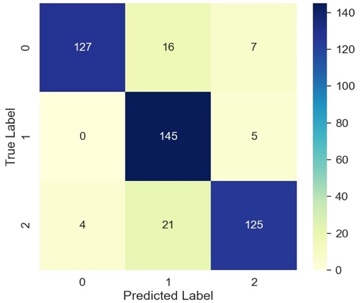 | 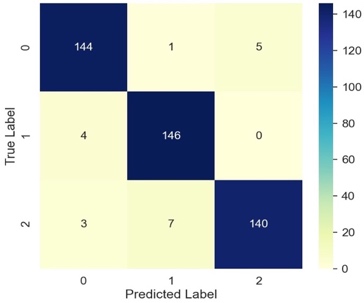 | 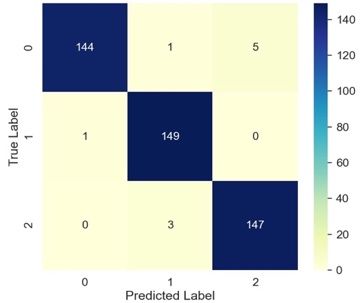 | 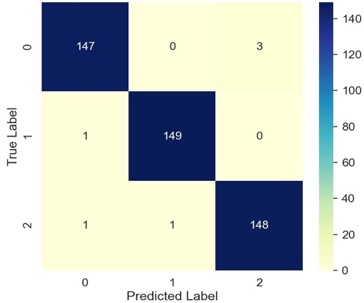 |
| --- | --- | --- | --- |
| (a) CapsNet | (b) IR-caps | (c) IRC-caps | (d) IRCM-Caps |

**ROC curve comparison**

| 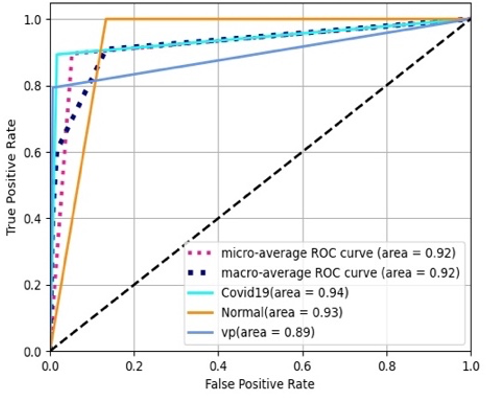 | 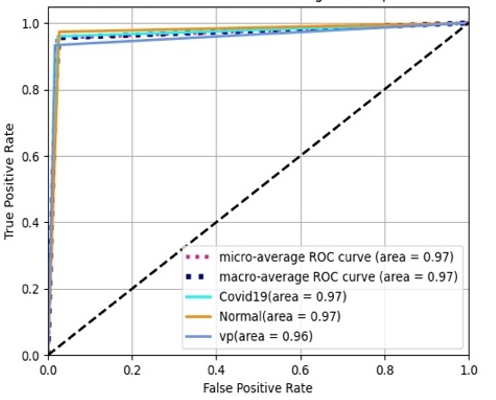 | 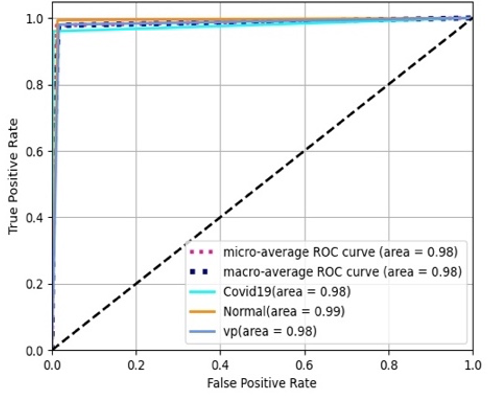 | 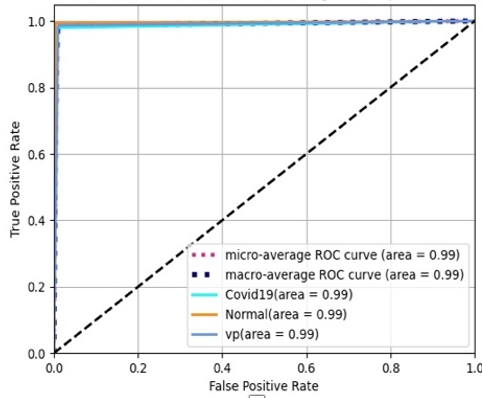 |
| --- | --- | --- | --- |
| (a) CapsNet | (b) IR-caps | (c) IRC-caps | (d) IRCM-Caps |
